# Case series: Immune checkpoint inhibitor-induced transverse myelitis

**DOI:** 10.3389/fneur.2023.1130313

**Published:** 2023-02-21

**Authors:** Sophie Chatterton, Shuo Xi, Jessica Xi Jia, Martin Krause, Georgina V. Long, Victoria Atkinson, Alexander M. Menzies, Suran L. Fernando, Thérèse Boyle, Samuel Kwok, Andrew Duggins, Deme Karikios, John D. E. Parratt

**Affiliations:** ^1^Department of Neurology, Royal North Shore Hospital, Sydney, NSW, Australia; ^2^Department of Neurology, Nepean Hospital, Sydney, NSW, Australia; ^3^Faculty of Medicine, University of Sydney, Sydney, NSW, Australia; ^4^Department of Medical Oncology, Royal North Shore Hospital, Sydney, NSW, Australia; ^5^Department of Oncology, Melanoma Institute Australia, Wollstonecraft, NSW, Australia; ^6^Department of Medical Oncology, Mater Hospital, Wollstonecraft, NSW, Australia; ^7^Department of Medical Oncology, Princess Alexandra Hospital, Brisbane, QLD, Australia; ^8^Clinical Immunology and Allergy Department, Royal North Shore Hospital, Sydney, NSW, Australia; ^9^NSW Health Pathology, Royal North Shore Hospital, Sydney, NSW, Australia; ^10^Department of Neurology, Westmead Hospital, Sydney, NSW, Australia; ^11^Department of Medical Oncology, Nepean Hospital, Sydney, NSW, Australia

**Keywords:** nivolumab, pembrolizumab, immune checkpoint, cancer, immune-related adverse event, transverse myelitis

## Abstract

**Introduction:**

Increasing implementation of the highly efficacious immune checkpoint inhibitors (ICIs) has raised awareness of their various complications in the form of immune-related adverse events (irAEs). Transverse myelitis following ICIs is thought to be a rare but serious neurologic irAE and knowledge is limited about this distinct clinical entity.

**Cases:**

We describe four patients across three tertiary centers in Australia with ICI-induced transverse myelitis. Three patients had a diagnosis of stage III–IV melanoma treated with nivolumab and one patient had stage IV non-small cell lung cancer treated with pembrolizumab. All patients had longitudinally extensive transverse myelitis on magnetic resonance imaging (MRI) spine and clinical presentation was accompanied by inflammatory cerebrospinal fluid (CSF) findings. Half of our cohort had received spinal radiotherapy, with the areas of transverse myelitis extending beyond the level of previous radiation field. Inflammatory changes on neuroimaging did not extend to the brain parenchyma or caudal nerve roots, except for one case involving the conus medullaris. All patients received high dose glucocorticoids as first-line therapy, however the majority relapsed or had a refractory state (3/4) despite this, requiring escalation of their immunomodulation, with either induction intravenous immunoglobulin (IVIg) or plasmapheresis. Patients in our cohort who relapsed had a poorer outcome with more severe disability and reduced functional independence following resolution of their myelitis. Two patients had no progression of their malignancy and two patients had malignancy progression. Of the three patients who survived, two had resolution of their neurological symptoms and one remained symptomatic.

**Conclusion:**

We propose that prompt intensive immunomodulation is favored for patients with ICI-transverse myelitis in an attempt to reduce associated significant morbidity and mortality. Furthermore, there is a significant risk of relapse following cessation of immunomodulatory therapy. We suggest one treatment approach of IVMP and induction IVIg for all patients presenting with ICI-induced transverse myelitis based on such findings. With the increasing use of ICIs across oncology, further studies are required to explore this neurological phenomenon in greater detail to help establish management consensus guidelines.

## Introduction

Over the last decade, immune checkpoint inhibitors (ICIs) have become well recognized as a novel therapy of great efficacy against a wide range of malignancies (1). With their advent, there has also been increasing acknowledgment of complications of ICIs in the form of immune-related adverse events (irAEs) (2). Neurological irAEs are thought to account for1–5% of all irAEs (3). Diagnosing neurological irAEs can be challenging due to their often subtle presentations and varying severities, and in the absence of prompt identification and implementation of treatment, can lead to significant disability or mortality. We describe four patients across Australia with ICI-induced transverse myelitis. To our knowledge, this is one of the largest case series published regarding this complication in ICIs.

## Case 1

A 52 year-old male presented with a 2 week history of rapidly ascending painful acral paraesthesia, mild lower limb weakness, back pain, bowel and bladder dysfunction and sensory ataxia. His main concern was his loss of functional independence given that prior to onset of his symptoms, he had been mobilizing unaided. His history was significant for resected stage IIIB melanoma (BRAF wildtype), hypertension, dyslipidaemia, non-alcoholic fatty liver disease and obesity for which he took perindopril and amlodipine. His initial melanoma included primary melanoma resected from the left shoulder in 2016, with an in-transit metastasis resected in October 2021. He was then commenced on adjuvant nivolumab (480 mg intravenously [IV] 4-weekly) in November 2021 for which he had received six cycles. He had developed immune-mediated hypothyroidism 2 months prior to presentation for which he was taking regular thyroxine supplementation, and mild dermatitis on regular emollients. An FDG-PET scan performed 1 day prior to admission had demonstrated no evidence of recurrence.

Physical examination revealed lower limb symmetrical hyperreflexia but otherwise normal tone, with no clonus and flexor plantar responses bilaterally. His power was normal aside from left hip flexion weakness 4+/5. There was evidence of loss of proprioception to the metatarsophalangeal joints and impaired distal temperature and pinprick sensation to the knees bilaterally. He had an ataxic gait.

**Figure 1:**
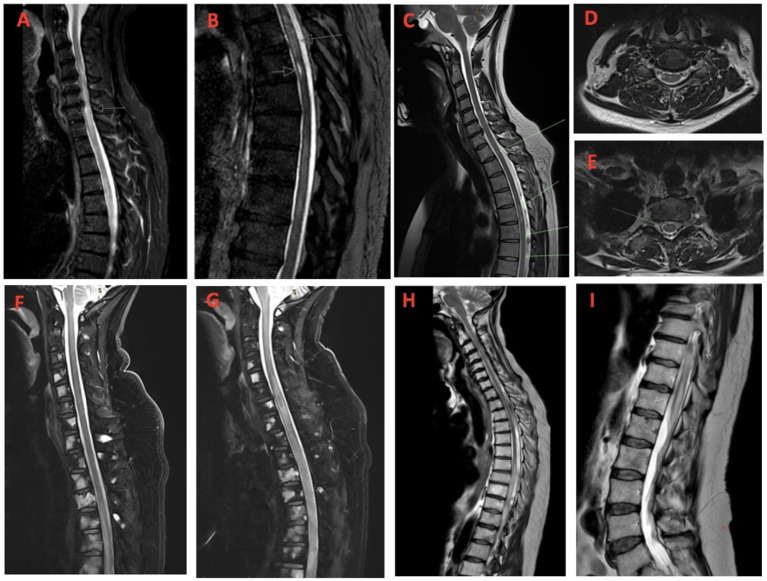
**(A, B)** Case 1-Sagittal MRI demonstrating eccentric patchy abnormal intramedullary high STIR signal and enhancement within the spinal cord, most conspicuous at the level of C6/C7 and T5 (arrows); **(C)** Case 2-Sagittal and **(D, E)** axial MRI images showing longitudinally extensive abnormal T2 signal hyperintensity in the cord from T4/T5 to T12 with a faint focus of cord signal hyperintensity at C5/C6 and at T1/2; **(F)** Case 3-Sagittal MRI images demonstrating longitudinally extensive abnormal T2 signal hyperintensity in the mid-thoracic cord on initial presentation and **(G)** extending to involve the mid- to lower cervical spinal cord 2 weeks later; **(H)** Case 4-Sagittal MRI images demonstrating longitudinally extensive abnormal T2 signal hyperintensity from mid-thoracic cord T6/T7, extending to the conus medullaris **(I)**.

MRI whole spine with gadolinium demonstrated patchy abnormal intramedullary high STIR signal and enhancement within the spinal cord, most conspicuous at the levels of C6/C7 and T5, suspicious for an inflammatory myelitis (Figures 1A, B). Nerve conduction studies (NCS) were unremarkable.

Cerebrospinal fluid (CSF) analysis was significant for a high protein level (1.78 g/L [reference range 0.20–0.60 g/L) and a mononuclear pleocytosis (mononuclear count 237 × 10^6^/L, polymorphonuclear cells [PMNs] 2 × 10^6^/L, red blood cell count [RBCs] 3 × 10^6^/L). CSF flow cytometry was non-diagnostic and cytology demonstrated numerous lymphocytes with no malignant cells. Serum myelin oligodendrocyte glycoprotein (MOG)-IgG was negative. Serum and CSF neuromyelitis optica (NMO)-IgG were not assessed. Serum and CSF antineuronal antibody panels (testing antibodies against PCA1, PCA2, Hu/ANNA-1, Ri/ANNA-2, Ma 1/2, amphiphysin, CV2 [CRMP-5], Tr, Sox-1 and NMDA receptor) were negative.

The patient was commenced on intravenous methylprednisolone 1 g once daily for 5 days with rapid improvement in his sensory symptoms and lower limb weakness with complete resolution of his back and neuropathic pain. His mobility improved over the 5 days with no ongoing ataxia and he was able to be discharged home 1 week later, on a weaning dose of prednisone (100 mg once daily for 3 days followed by a slow wean thereafter). Interval spinal MRI 1 month later demonstrated improvement of his spinal cord ill-defined T2 hyperintensities with ongoing enhancement.

Following reduction of his prednisone from 15 to 10 mg 2 months later, the patient developed recurrent numbness in both feet without motor disturbance, and neuroimaging demonstrated extension of his longitudinal myelitis. He was administered induction dosing of intravenous immunoglobulin (IVIg) 2 g/kg over 5 days followed by 1 g/kg over 2 days which was continued as monthly maintenance therapy, and his prednisone was increased back to 15 mg daily. Again, his symptoms resolved over the following 2 days and MRI 6 weeks later demonstrated much improved spinal cord T2 hyperintensity. He remains on monthly IVIg, 10 mg prednisone and is currently 4 months post discharge from hospital. Immunotherapy has been permanently discontinued with no recurrence of malignancy.

**Figure 2:**
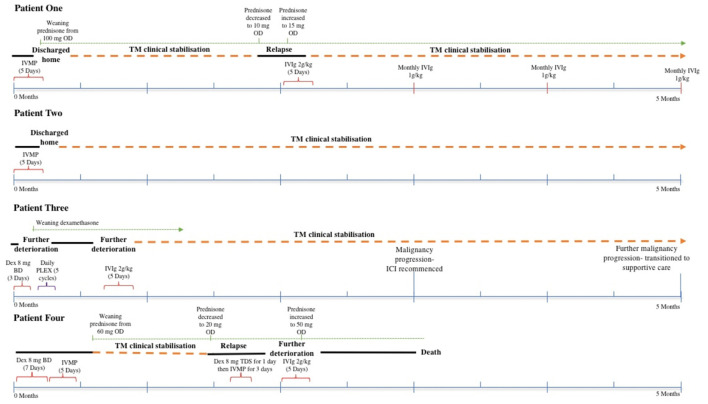
Timeline of each patient presented (IVMP, intravenous methylprednisolone; OD, once daily; TM, transverse myelitis; IVIg, intravenous immunoglobulin; BD, twice daily; PLEX, plasmapheresis; ICI, immune checkpoint inhibitor; TDS, three times daily).

A summary of each case and timeline has been provided in Table 1 and Figure 2.

**Table 1:** Comparison of the clinical manifestations, investigations and prognosis for each patient.

	**Patient 1**	**Patient 2**	**Patient 3**	**Patient 4**
Age at time of myelitis onset	52	40	47	64
Sex	Male	Female	Male	Male
Malignancy/mutation status/year of initial diagnosis	Stage IIIB melanoma (BRAF wildtype, 2016)	Stage IV melanoma (BRAF wildtype, 2016)	Stage IV melanoma (BRAF wildtype, 2022)	Stage IV non-small cell lung cancer (2021)
Immune Checkpoint Inhibitor received	Adjuvant nivolumab q4w	Nivolumab q4w	Combination nivolumab + ipilimumab q4w	Pembrolizumab 200 mg q3w
Spinal radiotherapy	No	No	Yes	Yes
Time from immune checkpoint inhibitor to onset of myelitis (months)	6	17	2	5
mRS pre-myelitis symptoms	0	0	0	2
Symptoms at presentation	Ascending distal numbness and paraesthesia, upper back pain, mild lower limb weakness, constipation, poor urinary stream, neuropathic pain, gait ataxia, lower limb weakness	Distal paraesthesia, Lhermitte's phenomenon	Distal numbness, paraparesis urinary retention	Lower back pain, ascending distal numbness and paraesthesia, lower limb weakness, fecal incontinence
CTCAE grade of neurological toxicity	3	2	4	5
Other irAEs	Immune-mediated hypothyroidism Immune-mediated dermatitis	Immune-mediated grade one xerostomia	Nil	Nil
MRI findings	Multiple patchy T2 hyperintensities, most prominent at C6/C7 and T5 with CE	Multiple patchy T2 hyperintensities throughout the thoracic cord with patchy CE	Longitudinally extensive T2 hyperintensity extending from mid- to lower cervical to lower thoracic spinal cord with initial CE	Longitudinally extensive central T2 hyperintensity extending from T6/T7 to conus medullaris, CE at T10/T11
CSF opening pressure (cm H_2_O)	N/A	↑ (28)	14 (normal)	N/A
CSF White Cell Count (WCC) (n/μL)	242 (237 mononuclears, 2 PMNs, 3 RBCs)	5 (5 mononuclears, 0 PMNs, 0 RBCs)	5 (WCC N/A, 148 RBCs)	4 (4 mononuclears, 0 PMNs, 360 RBCs)
CSF protein level (g/L)	↑ (1.78)	N (0.35)	↑ (0.9)	↑ (0.78)
CSF IgG/albumin ratio (%)	Normal	Normal	Borderline elevated	N/A
CSF-restricted OCB	No	No	Yes	N/A
mRS at time of peak symptoms	2	1	4	4
First-line therapy	IVMP 1 g OD (5 days)	IVMP 1 g OD (5 days)	Dexamethasone 8 mg BD followed by slow wean and PLEX for 5 daily exchanges	Dexamethasone 8 mg BD for 7 days, followed by IVMP OD for 5 days (1 g 1/7, 500 mg 2/7, 250 mg 2/7)
Relapse of myelitis (weeks later)	Yes (after 8 weeks)	No	Yes (after 2 weeks)	Yes
Second-line therapy	IVIg (2 g/kg induction dosing over 5 days, followed by maintenance 1 g/kg over 2 days every 4 weeks)	N/A	IVIg (2 g/kg induction dose over 5 days)	Dexamethasone 8 mg TDS for 1 day, followed by IVMP 1 g for 3 days, then IVIg (2 g/kg induction dose over 5 days) and weaning dose prednisolone
mRS at latest follow-up	1	0	4	6
Patient outcome	Complete resolution of symptoms.	Complete resolution of symptoms.	Motor and sensory level at C7. Palliative pathway.	Deceased
Malignancy status post immune checkpoint inhibitor	No recurrence	No progression. Complete response.	Progression of disease	Progression of disease
Malignancy treatment post-event	Remains off treatment for malignancy	Remains off treatment for malignancy	Received nivolumab for further 2 cycles complicated by further disease progression. Subsequent decision for best supportive care.	Off treatment for malignancy. After relapse of the TM, patient transitioned to best supportive care
Length of follow-up to date	4 months	10 months	9 months	3 months

CTCAE, Common Terminology Criteria for Adverse Events; PMNs, polymorphonuclear leukocytes; mRS, modified Rankin Scale; N/A, not available; OCB, oligoclonal bands; RBCs, red blood cells; IVMP, intravenous methylprednisolone; OD, once daily; IVIg, intravenous immunoglobulin; CE, contrast enhancement; BD- twice daily; PLEX, plasmapheresis; q4w- q4weekly; q6w- q6weekly.

## Case 2

A 40 year-old female presented with 5 weeks of acral paraesthesia and Lhermitte's phenomenon. There was no associated weakness and her bowel and bladder function was normal. Her background included previous thyroidectomy due to a multinodular goiter, well-controlled hypoparathyroidism and depression. Regular medications included thyroxine, calcium, calcitriol and sertraline. Notably she had stage IV melanoma (BRAF wildtype) with sites of disease including her right thigh, lung and lymph nodes. She had been commenced on nivolumab (480 mg IV 4-weekly) and had received seventeen cycles thus far. Her treatment had otherwise been complicated by grade one xerostomia. Her most recent imaging had shown a partial response with treatment.

Physical examination was normal with no objective sensory deficit identified. MRI whole spine demonstrated abnormal T2 signal hyperintensity in the spinal cord from T4/5 to T12 with patchy associated enhancement from T4/5 to T6, in keeping with an inflammatory demyelinating etiology (Figures 1C–E). A faint focus of cord signal hyperintensity at C5/6 and T1/2 could also be seen, without enhancement. No leptomeningeal enhancement was evident.

NCS and EMG which were normal. CSF opening pressure was elevated at 28 cm H_2_O. CSF protein was normal at 0.35 g/L and there was a mild mononuclear pleocytosis (5 × 10^6^/L mononuclear cells, 0 PMNs and 0 RBCs). CSF flow cytometry was non-diagnostic and cytology demonstrated a mild lymphocytosis with no malignant cells. Serum myelin oligodendrocyte glycoprotein (MOG)- and neuromyelitis optica (NMO)-IgG were both negative. Antineuronal antibodies were not assessed.

The patient proceeded to have intravenous methylprednisolone 1 g once daily for 5 days without an oral corticosteroid taper, followed by prompt symptom improvement over the following 2 months and then more gradual complete symptom resolution after 6 months. Immunotherapy was permanently discontinued. Progress imaging 3 months following steroid administration demonstrated that the previous focal areas of cord T2 signal hyperintensity in the mid to lower thoracic cord were much less apparent, and the areas of patchy enhancement in the mid thoracic cord had resolved. Progress imaging 9 months following steroid therapy demonstrated even further improvement with the lesions being essentially non-discernible. She remains well with evidence of complete response of her melanoma to therapy.

## Case 3

A 47 year-old male presented with concerns of an acute painful acral paraesthesia with lower limb weakness and urinary retention. He had a background of long standing inactive Crohn's disease on ustekinumab (an interleukin[IL]-12 and IL-23 inhibitor), and more recently, stage IV melanoma (BRAF wild-type) with known symptomatic vertebral metastases. He had received two cycles of combination ipilimumab and nivolumab (480 mg IV 4-weekly) as well as radiotherapy to T4–T8 for his symptomatic bone metastases (20 Gy in 5 fractions).

Physical examination revealed marked lower limb weakness and sensory examination was normal to all modalities. MRI spine demonstrated extensive bony metastatic disease with no evidence of fracture or cord/nerve root impingement, in addition to an ill-defined cord signal T2 hyperintensity with contrast enhancement in the mid-thoracic spine that was initially thought to be related to recent radiotherapy or cord metastasis (Figure 1F). He was commenced on dexamethasone 8 mg twice daily but 3 days later he deteriorated, developing a paraparesis and evidence of a sensory level at the mid-abdomen, and the possibility of transverse myelitis was raised.

The following day a lumbar puncture was performed which was significant for elevated protein (0.9 g/L) and a mild pleocytosis (WCC 5 x 10^6^/L). Cytology demonstrated scattered lymphoid cells but no malignant cells. CSF IgG/albumin ratio was borderline elevated at 24% (reference range 8–23%) and CSF-restricted oligoclonal bands were detected. CSF NMO- and MOG-IgG were both negative. Serum and CSF antineuronal antibody panels were also negative. An FDG-PET scan demonstrated marked metabolic regression of his skeletal metastases and no FDG-avid intraspinal metastases. He was diagnosed with checkpoint inhibitor-induced transverse myelitis.

The patient continued to deteriorate with escalating neuropathic pain and a rapidly ascending sensory level to T1, and a decision was made to escalate therapy to plasmapheresis. He proceeded to have five daily exchanges with improvement in his neuropathic pain after 2 days but no change in his focal neurological deficit. Following plasmapheresis over the subsequent week his symptoms continued to worsen with extension of his sensory level to C7 and complete paralysis below the C7 level. Progress MRI demonstrated progression of demyelination with longitudinal extension of T2 signal hyperintensity from the T12 level into the mid to lower cervical spinal cord. No cord enhancement however could be seen post contrast (Figure 1G).

Following clinical and radiological deterioration the patient proceeded to have intravenous immunoglobulin induction therapy (2 g/kg over 5 days) with stabilization of his symptoms and examination findings. Despite stabilization the patient continued to have a residual C7 sensory level and complete paralysis below C7. He was subsequently transferred to rehabilitation 3 weeks later and immunotherapy was ceased. He remained on dexamethasone with a gradual taper over 5 weeks from the time of initial presentation.

At rehabilitation, 2 months following his initial presentation, he underwent a progress FDG-PET/CT which demonstrated increasing size and metabolic activity within at least two liver and multiple skeletal metastases, consistent with disease progression. In discussion with the patient and his family regarding supportive care vs. ongoing treatment, the patient elected to be treated and he was offered a rechallenge of nivolumab monotherapy. With recommencement of nivolumab monotherapy he had no deterioration in his neurological deficit or other autoimmune toxicity. A further 2 months later he had another progress FDG/PET which demonstrated an increase in size and activity of the multiple bone and liver metastases. Nivolumab was discontinued and he was referred to palliative care 6 months after initial presentation.

## Case 4

A 64-year-old male presented to hospital with 5 weeks of progressively worsening lower back pain, lower limb weakness, ascending limb paraesthesia and bowel and bladder dysfunction. The patient had a background of type 2 diabetes mellitus, ischaemic heart disease, chronic obstructive pulmonary disease, osteoarthritis, and was an active smoker. He had stage IV non-small cell lung cancer with bone and lymph node metastases, and had received palliative radiotherapy to his whole cervical spine (20 Gy in 5 fractions), right iliac crest and femur shortly following diagnosis in October 2021. This was followed by four cycles of carboplatin/pemetrexed with partial response in lungs and mediastinal lymph nodes and stable bone metastases. He was then commenced on maintenance pembrolizumab (200 mg every 3 weeks) from February 2022 and he had received seven cycles in total prior to his symptom onset. Pre-morbidly, he occasionally required crutches for mobility at home due to severe hip osteoarthritis, but otherwise he was independent with activities of daily living.

Initial physical examination was significant for severe lower limb weakness, in addition to loss of pin prick sensation in the right T11/T12 dermatome and to the level of the ankle on the left. Proprioception was intact throughout the right lower limb, but was impaired on the left side to the ankle. Anal tone was intact.

An MRI whole spine demonstrated extensive central high T2 signal within the spinal cord from T6/T7 level to the conus medullaris, with evidence of contrast enhancement at T10/T11 (Figures 1H, I). CSF analysis demonstrated elevated protein (0.78 g/L) however only 4 WCCs were identified (all mononuclear cells). No malignant cells were seen and flow cytometry was non-diagnostic. CSF NMO and MOG-IgG were both negative. Serum and CSF antineuronal antibody panels were also negative.

The patient was initially treated with seven days of oral dexamethasone 8 mg twice daily, followed by 5 days of intravenous methylprednisolone (1 g initially then 500 mg for 2 days then 250 mg for 2 days), with almost complete resolution of lower limb weakness over the following week. He was discharged home 2 weeks after presentation on a tapering dose of prednisolone (from 60 mg/day with weekly wean by 10 mg over 6 weeks).

Four weeks after being discharged from hospital when his prednisolone had been weaned to 20 mg daily, he had re-emergence of his symptoms over a seven day period. Examination re-demonstrated severe lower limb weakness and reduced pinprick sensation to T12 dermatomal level with intact proprioception in the lower limbs bilaterally. Repeat MRI spine demonstrated ongoing longitudinally extensive transverse myelitis, with larger left T8/9 and right T12 paravertebral soft tissue deposits. He also proceeded to have a repeat CT chest/abdomen/pelvis, which revealed progression of his metastatic disease, including new bilateral adrenal masses and abdominal para-aortic lymphadenopathy.

The patient was initially treated with dexamethasone 8 mg TDS for 1 day and then was changed to IV methylprednisolone 1 g daily for 3 days. This time he had a poor clinical response and so IVIg 2 g/kg was administered over 5 days with a concurrent weaning dose of high dose oral prednisolone 50 mg/day (reduced by 12.5 mg daily per week). He continued to show no clinical improvement with progressive worsening of his paraparesis and urinary incontinence and his goals of care were changed to best supportive care. The patient died within 3 weeks.

## Discussion

The advent of immunotherapy has revolutionized the treatment of many cancers, through its mechanism of stimulating the immune system to aide detection and eradication of malignant cells (1). Nivolumab and pembrolizumab function as IgG4 subtype monoclonal antibodies against programmed cell death protein 1 (PD-1) receptor, a crucial inhibitory receptor expressed by activated T and B lymphocytes which normally act to mediate the immune response by downregulating effector functions and inhibiting the development of immune memory (4–6). PD-1 is activated by ligand PD-L1, which is expressed by tumor cells and infiltrating immune cells, and inhibition of this interaction between the PD-1 receptor and ligand can enhance anti-tumor immunity, resulting in durable tumor control (4).

With the rising implementation of ICIs there has been increased recognition of their unique secondary autoimmune complications through activation of the immune system (7). Inhibition of checkpoint proteins such as PD-1 and CTLA4 triggers lymphocytic activation and enhances recognition and destruction of tumor cells (7). As a result of an enhanced immune response, ICI may cause a variety of autoinflammatory disorders in susceptible individuals, known as immune related adverse events (irAEs). Neurologic irAEs are estimated to occur in up to five percent of patients with ICIs and clinical manifestations can include autoimmune encephalitis, Gullian-Barré-like syndrome, motor/sensory and autonomic peripheral neuropathies, myasthenia gravis-like syndrome, posterior reversible encephalopathy syndrome, aseptic meningitis and transverse myelitis (3, 7). Whilst neurological irAEs represent a relatively small proportion of all reported irAEs with ICIs, they embody a heterogeneous group that can often present insidiously, have a vast array of differential diagnoses (including progressive malignancy) and can result in severe disability or death, and therefore are perhaps the most challenging irAEs to detect and treat. Furthermore, data regarding treatment recommendations remains very limited for neurologic irAEs and recommendations typically arise from isolated case reports (7).

Transverse myelitis following ICIs is thought to be an uncommon but potentially catastrophic neurologic irAE (8). To our knowledge, there is only one other case series of transverse myelitis associated with ICIs in 7 patients treated with either anti-PD1 alone (*n* = 6) or in combination with anti-CTLA4 (*n* = 1) (8). Similar to this series, our patients all had longitudinally extensive myelitis on MRI and clinical presentation was accompanied by inflammatory CSF findings with a mild pleocytosis and typically moderate CSF protein elevation. Similar to their cases where 3/7 patients had received thoracic radiotherapy, half of our cohort (2/4) had also received spinal radiotherapy. In contrast to the previous study however, inflammatory changes in our patients did not extend to the brain parenchyma or caudal nerve roots on neuroimaging, except for one case involving the conus medullaris (8).

Similar to other case reports and as highlighted in the case series (8), we found a high rate of relapse or refractory disease (75%) with initial therapy with corticosteroids. Patients in our cohort who relapsed had a poorer outcome with more severe disability and reduced functional independence following resolution of their myelitis. A variety of modalities have been utilized but we demonstrated consistent benefit from IVIg or plasmapheresis in addition to corticosteroids. Moreover, these therapies were well tolerated with no major adverse events reported. These observations support the notion that prompt intensive immunomodulation (IVIg or plasmapheresis) in addition to corticosteroids is favored for patients with ICI-transverse myelitis in an attempt to significantly reduce associated morbidity.

Proposed reasons for relapse is that the pathogenesis may involve the activation of dormant autoimmune diseases by clonal expansion of pre-existing self-reactive T-lymphocytes and/or the induction of paraneoplastic processes through the initiation or exacerbation of immune responses against shared autoantigens between tumor cells and neural tissue (9). While this requires further elucidation in longitudinal studies it would be likely that these processes would require long-term treatment with adjuvant immunomodulation. Furthermore, the half-lives of nivolumab and pembrolizumab, respectively are 27 and 26 days, similar to that of endogenous immunoglobulin (10). Previous case series studying the delayed irAEs after nivolumab revealed that nivolumab was persistent in the serum for months at a concentration sufficient to induce irAEs (11). Delayed irAEs associated with ICI and the high relapse rates seen in our case series, suggests that cautious tapering of immunomodulatory therapy is recommended in neurological irAE to reduce recurrence of symptoms.

Current guidelines recommend permanent discontinuation of ICI with grade 3 or 4 irAEs including all neurological AEs (12). A retrospective study of recurrence of irAE from rechallenge with the same ICI after a first irAE using a pharmacovigilance database discovered that life-threatening irAE such as myocarditis and neurological complications were not associated with a higher recurrence rate than other irAE, with neurological irAE recurring in only 3 out of 19 patients (13). Resuming ICI could be considered in select populations with sustained resolution of their neurological complications (14).

In Cases 3 and 4, the area of spinal T2 hyperintensity extended beyond the level of previous radiation field. This could suggest that radiotherapy may augment susceptibility to ICI-induced myelitis, potentially due to disruptions in the blood brain barrier through mechanisms of endothelial cell death and surrounding microglial and astrocyte neuro-inflammatory response (15). While the total spinal radiotherapy dosage in each case was well below the toxic threshold for radiation induced myelopathy, which makes radiation induced necrosis of the spinal cord less likely, it is possible that there is a synergistic effect by which radiotherapy and ICI exert their antitumour effect, potentially increasing the risk of neurological irAE (16). To our knowledge there are four previously reported cases of combined ICI and radiotherapy-related myelitis, with variable outcomes (17–20), with one case successfully treated with rechallenge of pembrolizumab without recurrence of irAE (17).

While our case series is limited by its small sample size and relatively short follow-up period, it emphasizes two key points. Firstly, it highlights the substantial morbidity and mortality associated with ICI-induced transverse myelitis and thus the need for prompt initial intensive immunomodulation. Secondly, our study demonstrates the significant risk of relapse of ICI-induced transverse myelitis following cessation of immunomodulation. We propose one treatment approach of intravenous pulse methylprednisolone and induction dose IVIg or plasmapheresis for all patients presenting with ICI-induced transverse myelitis, followed by a slow corticosteroid wean, based on such findings. With the increasing use of ICIs as a cornerstone of cancer therapy, further studies are required to explore this neurological phenomenon in greater detail to help establish consensus guidelines on its management.

## Data availability statement

The original contributions presented in the study are included in the article/supplementary material, further inquiries can be directed to the corresponding author.

## Ethics statement

The studies involving human participants were reviewed and approved by Northern Sydney Local Health District Human Research Ethics Committee. The patients/participants provided their written informed consent to participate in this study.

## Author contributions

Conceptualization and supervision: MK and JP. Writing—original draft: SC, SX, JJ, and VA. Writing—review and editing: SC, SX, JJ, MK, GVL, VA, AM, SF, TB, SK, AD, DK, and JP. All authors contributed to the article and approved the submitted version.
